# Effects of Coronavirus Disease Pandemic on Tuberculosis Notifications, Malawi

**DOI:** 10.3201/eid2707.210557

**Published:** 2021-07

**Authors:** Rebecca Nzawa Soko, Rachael M. Burke, Helena R.A. Feasey, Wakumanya Sibande, Marriott Nliwasa, Marc Y.R. Henrion, McEwen Khundi, Peter J. Dodd, Chu Chang Ku, Gift Kawalazira, Augustine T. Choko, Titus H. Divala, Elizabeth L. Corbett, Peter MacPherson

**Affiliations:** Malawi Liverpool Wellcome Clinical Research Programme, Blantyre, Malawi (R. Nzawa Soko, R.M. Burke, H.R.A. Feasey, W. Sibande, M. Nliwasa, M.Y.R. Henrion, M. Khundi, A.T. Choko, T.H. Divala, E.L. Corbett, P. MacPherson);; London School of Hygiene and Tropical Medicine, London, UK (R. Nzawa Soko, R.M. Burke, H.R.A. Feasey, M. Khundi, T.H. Divala, E.L. Corbett, P. MacPherson);; University of Malawi College of Medicine, Blantyre (M. Nliwasa);; Liverpool School of Tropical Medicine, Liverpool, UK (M.Y.R. Henrion, P. MacPherson);; University of Sheffield, Sheffield, UK (P.J. Dodd, C.C. Ku); District Health Office, Blantyre (G. Kawalazira)

**Keywords:** respiratory infections, severe acute respiratory syndrome coronavirus 2, SARS-CoV-2, SARS, COVID-19, coronavirus disease, zoonoses, viruses, coronavirus, tuberculosis, bacteria, disease surveillance, health systems

## Abstract

The coronavirus disease (COVID-19) pandemic might affect tuberculosis (TB) diagnosis and patient care. We analyzed a citywide electronic TB register in Blantyre, Malawi and interviewed TB officers. Malawi did not have an official COVID-19 lockdown but closed schools and borders on March 23, 2020. In an interrupted time series analysis, we noted an immediate 35.9% reduction in TB notifications in April 2020; notifications recovered to near prepandemic numbers by December 2020. However, 333 fewer cumulative TB notifications were received than anticipated. Women and girls were affected more (30.7% fewer cases) than men and boys (20.9% fewer cases). Fear of COVID-19 infection, temporary facility closures, inadequate personal protective equipment, and COVID-19 stigma because of similar symptoms to TB were mentioned as reasons for fewer people being diagnosed with TB. Public health measures could benefit control of both TB and COVID-19, but only if TB diagnostic services remain accessible and are considered safe to attend.

Tuberculosis (TB) is a major killer, causing ≈1.4 million deaths worldwide annually (*1*), making it second only to coronavirus disease (COVID-19) as the biggest cause of infectious disease deaths in 2020 (*2*). In addition to the direct health effects of COVID-19, the secondary effects of the COVID-19 pandemic, including lockdowns, economic turmoil, healthcare worker illness and attrition, overwhelmed health facilities, and fear of healthcare facilities, might affect delivery of health services (*3*). Concerns have been raised that COVID-19 could adversely affect TB disease diagnosis, treatment, and prevention, reversing recent progress in improving TB case detection and reducing deaths, although protective measures used for COVID-19 also could reduce TB transmission (*1*,*3*,*4*). Initial modeling published in May 2020 suggested that healthcare service disruption worldwide could lead to 6.3 million additional TB cases and 1.4 million additional TB deaths from 2020 through 2025 because of TB underdiagnosis and interruptions in TB treatment (*5*). Empirical data from settings with high TB burdens are urgently needed to examine the effects of COVID-19 on TB and to determine mitigation strategies (*4*).

According to the World Health Organization, Malawi is 1 of 30 countries that have high TB and HIV burdens (*1*). In Blantyre, in the southern region of Malawi, a citywide electronic TB register has been maintained in partnership by the Malawi Liverpool Wellcome Trust, Malawi National Tuberculosis Programme, and Blantyre District Health Office (*6*). We used these data to investigate the effects of COVID-19 on citywide TB case notifications. We hypothesized that the direct and indirect effects of the COVID-19 epidemic in Malawi would reduce TB case notifications and that effects might have been experienced disproportionately at different health system levels and by certain population groups, including persons living with HIV. Our primary objective was to estimate the number of missed TB case notifications. Our secondary objective was to determine whether missed notifications were affected by sex, health facility, or HIV status. Finally, to investigate and explain the underlying causes of under notification of TB, we performed a qualitative study with TB officers, the cadre of healthcare workers who provide most TB services in Malawi.

## Methods

### Data Sources

To estimate population denominators for Blantyre District, we obtained age- and sex-specific background mortality rates and fertility rates from 2008–2020 World Population Prospect data (*7*). We used the cohort-component method to combine these data into local estimates from the 2008 and 2018 Malawi national population censuses.

In Blantyre, TB officers working at all primary health centers and the city’s main hospital, Queen Elizabeth Central Hospital (QECH), record demographic and clinical characteristics of all TB patients who register for treatment by using an electronic case record form. Data collected includes date and clinic of registration, age, sex, HIV status, residential address, and TB characteristics, such as pulmonary versus extra-pulmonary TB and microbiological classification. Records are reconciled with the Ministry of Health National Tuberculosis Programme treatment registers every quarter. Each month, a randomly selected 5% sample of people who registered for TB treatment undergoes home tracing for data validation purposes.

### Statistical Modeling

To investigate the effects of COVID-19 on TB case notification in Blantyre, we conducted an interrupted time series analysis (*8*). The Malawi government declared a state of emergency because of COVID-19 on March 23, 2020, and the first COVID-19 cases were diagnosed on April 2, 2020. We assumed that COVID-19 restrictions and the government and public response to the emerging epidemic would cause both an immediate step change in TB case notifications and a slope change leading to different month-by-month trends than those seen before COVID-19 (*8*). Using a negative binomial distribution to account for overdispersion, we modeled monthly counts of TB cases as a function of month, COVID-19, and month-given-COVID-19, with an offset term to account for underlying population (Appendix). We used TB notification data from June 2016, when the country began a universal test-and-treat program to provide antiretroviral therapy for persons with HIV and started using the Xpert MTB/RIF assay (*9*), which rapidly diagnoses *Mycobacterium tuberculosis*, the bacterium that causes TB disease, and rifampin resistance in <2 hours (*10*).

We estimated trends in TB case notification rates (CNRs) by using estimated Blantyre census population denominators to convert model-fitted monthly numbers of notified cases to annualized equivalent cases per 100,000 population. We used the model to predict TB CNRs from April 2020 on under a counterfactual situation in which COVID-19 had not occurred and background trends from April 2016 and March 2020 continued linearly. We defined numbers of missed TB cases as the difference between the observed numbers of notified cases and numbers expected under the counterfactual no–COVID-19 situation, acknowledging that some of the missed cases might be diagnosed later and thus be delayed rather than entirely missed. We estimated the 95% CI for the total number of missed TB cases through 1,000 parametric bootstrap replications. We took observed cases as-is and predicted cases under the counterfactual scenario from a normal distribution on the link scale with the mean equal to model prediction for given month under the counterfactual and SD equal to model SE for predictions for the given month under the counterfactual scenario.

For the secondary objective, we modeled the differential effect of COVID-19 on TB case notifications by sex, HIV status, and whether TB was diagnosed at the QECH or primary care level (Appendix). Because a small amount of data were missing for HIV status and sex, we performed multiple imputations using chained equations with predictive mean matching by using the mice package in R software (*11*).

All decisions about the expected effect model (i.e., a step and slope change), the date of change (i.e., April 2020), and the covariates in model 2 (i.e., age, sex, and primary care vs. QECH) were made a priori on the basis of knowledge about likely effects of COVID-19 and covariates known to differentially affect access to TB healthcare (*12*). To assess the statistical significance of the change in TB notifications concurrent with COVID-19 epidemic in Malawi, we extracted residuals from a regression that did not model changes due to COVID-19. We compared the sum of the residuals for the 9 months during the COVID-19 epidemic in Malawi, April–December 2020, with the distribution of this statistic from 1 million randomly permuted residuals. We also computed this statistic for all 9-month windows, excluding COVID-19 within the data.

### Sensitivity Analysis

TB exhibits seasonality related to climate and weather conditions (*13*). Therefore, we performed a sensitivity analysis by adding seasonal effects to the interrupted time series model by using a harmonic term with 2 peaks every 12 months.

### Qualitative Analysis

During October 21–December 14, 2020, we conducted in-depth interviews with 12 TB officers from healthcare facilities in Blantyre, 2 from QECH and 10 from primary healthcare centers, to ascertain the main reasons for changes in TB case notifications during the COVID-19 pandemic. A local social scientist with experience of qualitative interviewing conducted interviews in Chichewa, the local language. Data were recorded and simultaneously transcribed and translated to English. We developed a thematic framework from the initial 4 interviews, which we applied across all subsequent interviews. Coding and data analysis were done using NVIVO (QSR International, https://www.qsrinternational.com). Interviews were continued until saturation of themes was reached. We did not interview persons attending clinics to receive healthcare.

### Ethics Approval

Participants provided oral consent for their data to be recorded in the enhanced surveillance dataset. A waiver of requirement for written consent was approved by London School of Hygiene and Tropical Medicine and College of Medicine, University of Malawi, both of which provided ethical approval for the Blantyre enhanced TB surveillance system and qualitative interviews. TB officer participants in the in-depth interviews provided informed written consent.

## Results

### Interrupted Time Series

During June 2016–December 2020, a total of 10,274 people starting TB treatment were notified in Blantyre. During June 2016–March 2020 (i.e., before COVID-19), annualized Blantyre TB CNRs fell by ≈1% per month, reaching a peak of 405 cases/100,000 persons in November 2016 and declining to 137 cases/100,000 persons in October 2019. A total of 9,199 TB cases were notified in Blantyre during the pre–COVID-19 period (June 2016 to December 2020), 3,561 among women and girls and 5,611 in men and boys; 27 cases were missing data on sex. Persons living with HIV represented 5,820 (63.3%) TB notifications and 3,279 (35.6%) HIV-negative persons were among notified TB cases; 100 TB cases had missing data or unknown HIV status. TB notifications were split almost evenly between QECH (4,889 notifications; 53.1%) and primary health facilities (4,310 notifications; 46.9%). Children <14 years of age comprised 920 (10%) notifications. The median age among adults with diagnosed TB was 35 (interquartile range [IQR] 28–44) years for women and 37 (IQR 30–45) years for men.

**Figure 1:**
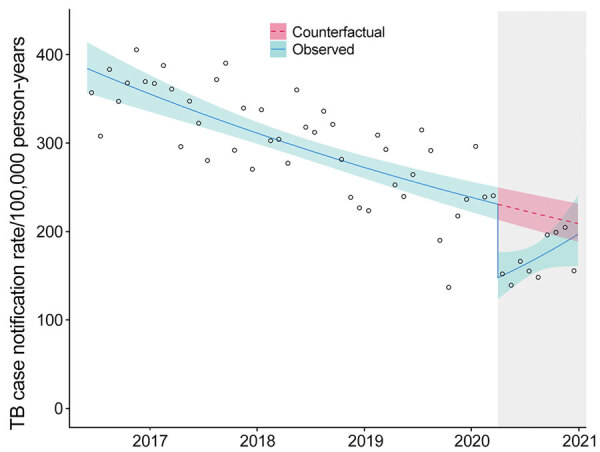
Effects of coronavirus disease (COVID-19) pandemic on monthly TB case notification rates in Blantyre, Malawi. Circles represent the observed number of cases each month. Solid blue line represents the fitted model with both step and slope change due to COVID-19; teal shaded area represents 95% CI. Pink dotted line represents counterfactual expected TB rates; pink shaded area represents 95% CI. Gray shaded area on the right indicates timeframe in which the COVID-19 emergency was declared in Malawi. TB, tuberculosis.

The declaration of a national COVID-19 disaster led to an abrupt 35.9% (95% CI 22.1%–47.3%) decline in TB notifications in April 2020 (Figure 1). However, subsequent TB notifications increased at a rate of 4.40% (95% CI 0.59%–8.36%) per month. The effect of the initial decline at the start of the COVID-19 pandemic was that observed Blantyre TB annualized CNRs pre–COVID-19, in March 2020, were 240 cases/100,000 persons and rates after the COVID-19 disaster declaration were 152 cases/100,000 persons in April 2020. By comparison, the predicted April CNR in the counterfactual scenario without COVID-19 was 230 cases/100,000 person-years. However, by November 2020, observed Blantyre TB CNRs were 205 cases/100,000 person-years and December 2020 rates were 156 cases/100,000 person-years, compared with a predicted CNR of 213 cases/100,000 person-years in November and 211 cases/100,000 person-years in December in the counterfactual scenario.

During April–December 2020, a total of 1,075 TB cases were notified in Blantyre, equivalent to 196 cases/100,000 person-years (Table 1). Under the counterfactual situation of no COVID-19 epidemic, we would expect 1,408 (95% CI 1,366–1,451) TB cases would have been notified, equivalent to annualized case notification rate of 221 cases/100,000 person-years. Therefore, we estimate that the COVID-19 epidemic directly and indirectly led to 333 (95% CI 291–376) fewer TB notifications, a 23.7% (95% CI 21.4%–26.0%) reduction in TB notifications.

**Table 1:** Modeled effects of coronavirus disease pandemic on tuberculosis case notifications, April–December 2020, Blantyre, Malawi*

Models	Observed no. notified TB cases with COVID-19	Median counterfactual model-estimated no. notified TB cases without COVID-19 (95% CI)		
			% Difference (95% CI)	
			Absolute	Relative
Model 1				
Overall	1,075	1,408 (1,366–1,451)	333 (291–376)	23.7 (21.4–26.0)
Model 2				
Sex				
M	692	875 (848–901)	183 (156–209)	20.9 (18.5–23.3)
F	383	553 (534–571)	170 (151–188)	30.7 (28.4–33.0)
Primary health centers	488	761 (737–785)	273 (249–297)	35.9 (33.9–37.9)
Queen Elizabeth Central Hospital	587	666 (645–688)	79 (58–101)	11.9 (9.10–14.7)
HIV status				
HIV-positive	660	820 (796–845)	160 (136–185)	19.6 (17.2–21.9)
HIV-negative	415	607 (586–627)	192 (171–212)	31.6 (29.3–33.8)

*COVID-19, coronavirus disease; TB, tuberculosis.

**Figure 2:**
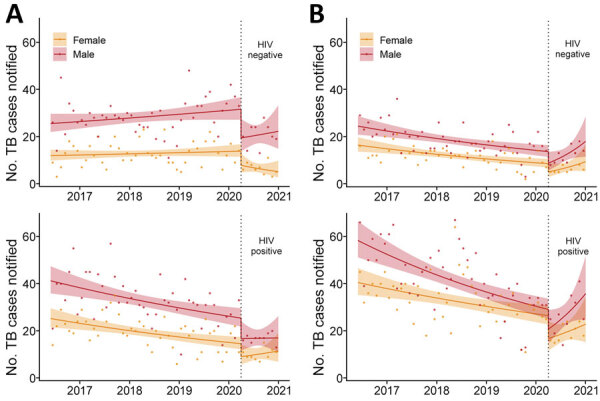
Effects of coronavirus disease (COVID-19) on monthly TB case notifications in Blantyre, Malawi, by HIV status, registration site, and sex. A) TB notifications at primary healthcare centers. B) TB notifications at Queen Elizabeth Central Hospital. Dots indicate observed number of cases per month. Solid lines indicate fitted model with both step and slope change due to COVID-19; shaded areas indicate 95% CI. Vertical dotted lines indicate time that COVID-19 emergency was declared in Malawi. TB, tuberculosis.

As a secondary objective, we modeled which population groups were most affected by disruption to TB services (Figure 2). This model incorporated sex, HIV status, and healthcare facility (QECH vs. primary care clinics) and estimated that 352 (95% CI 319–385) TB cases were missed during April–December 2020. Men and boys accounted for a slightly larger number of missed TB diagnoses with 183 (95% CI 158–209) missed cases compared with 170 (95% CI 151–188) missed cases among women and girls. However, women and girls had a larger proportional decline, 30.7% (95% CI 28.4%–33.0%) than did men and boys, 20.9% (95% CI 18.5%–23.3%). Notifications at primary healthcare centers also were disproportionately reduced compared with hospital notifications, as were notifications for HIV-negative persons compared with those living with HIV (Table 2). The nonoverlapping confidence intervals for these groups indicated statistically significant differences in effects of COVID-19 by gender, HIV status, and healthcare setting.

**Table 2:** Quotations from in-depth interviews with health officers about reasons for reduced tuberculosis notifications due to coronavirus disease epidemic in Malawi, June–December 2020*

Theme, quote no.	Participant no., sex	Quote
Fear of COVID-19 contagion at health facilities		
Q1	02, F	“People were afraid of getting infected if they come to the facility.”
Q2	09, F	“… they were afraid saying that if the workers are found with COVID, so if we go there they will infect us.”
COVID-19 related health facility closures		
Q3	03, F	“…they were told that the clinic had been shut down and people are not being assisted… which means people were just staying in their homes and the TB was just being spread amongst them.”
Q4	02, F	“Our facility wasn’t closed, but there was a certain week that we were just going but we were not working because there was no PPE, so people were afraid. There were no gloves, no masks how were we going to work? So a sit-in happened.”
Q5	05, F	“Yes we had a strike at this hospital and the strike occurred in all health centres. The reason behind the strike was that COVID-19 was at its peak but we didn’t have PPE which was putting us at risk.”
Q6	07, M	“The first strike was against shortage of PPEs and the second strike was organized by Interns who were complaining that they are making them work on this dangerous disease of COVID-19 yet they are not being employed… And the other strike was about risk allowance.”
Effects of COVID-19 prevention measures on healthcare access		
Q7	04, F	“…then government announced that wearing of mask is mandatory some people who couldn’t manage wear the mask were making a decision of not going to the hospital instead, some were complaining that they suffocate in a mask.”
Q8	08, F	“…all patients should be wearing masks when coming here but some patients were ignoring and when we send them back to go and get a mask some patients were ending up not coming back.”
Q9	08, F	“Some people travel from far communities to come here and the increase in transport fare also influenced some people to fail to come to the hospital.”
Similarity of TB and COVID-19 symptoms leading to reduced access to TB care		
Q10	02, F	“… sometimes they think that if they test positive [for COVID-19], people will discriminate them, they have fear of unknown. So during this period people weren’t coming to say I have a cough, test me, they were just staying at home buying Bactrim and drinking it at home.”
Q11	06, F	“the signs and symptoms of COVID-19 and TB were somehow similar so because the signs were similar people were scared to come to the hospital because they were assuming that instead of testing them for TB we will test them for COVID-19”
Q12	07, M	“They were communicating that if a person has fever then that is a sign of COVID-19 and that particular person is required to go into isolation so people were afraid to come to the hospital when they have fever because of the messages that they may be isolated with their families.”
Q13	01, M	“… they were expecting that someone who has COVID-19 coughs and sneezes severely, and has fever and headaches, so when they ask about those, the same things that a TB patient presents, that was when those people were being sent back to go home and call the COVID-19 help line.”
Reduced healthcare worker capacity to support TB testing		
Q14	05, F	“… we were no longer asking many questions once the person tells us that she has dry cough we were running away from that person… Because if the person has dry cough the first thing that we were thinking of is COVID-19.”
Q15	11, F	“I was scared because it was difficult to know if the patient is coughing because of TB or COVID-19.”
Q16	01, M	“… in the laboratory… the ones that are involved in the testing, they were refusing to handle sputum because they were taught that sputum has the highest concentration of COVID-19 so some were dodging which was resulting in delays.”

*COVID-19, coronavirus disease; PPE, personal protective equipment; TB, tuberculosis.

The drop in TB notifications during April–December 2020 was greater than that for any other 9-month period observed, and the sum of the residuals during this period was more negative than expected by random chance (p = 0.004). The sum of residuals in other 9-month periods was significantly more negative than anticipated from random resampling (p<0.05), indicating a unique statistically significant drop in cases during April–December 2020. Sensitivity analysis around seasonality of TB did not materially affect the conclusions.

### Qualitative Results

Of the 12 in-depth interviews with healthcare providers, 9 participants were female and 3 were male; ages were 34–53 years. Most (10/12) participants had secondary-level education. Themes that emerged from the in-depth interviews related to both an overall reduction in persons attending health facilities and to TB-specific issues.

#### Reduced Attendance at Healthcare Facilities

In addition to reduced attendance at healthcare facilities among the general public from fear of being infected with COVID-19, participants mentioned that several healthcare workers tested positive for COVID-19 during the epidemic (Table 2). The facility-based COVID-19 outbreaks led to temporary closures for disinfection. Facility closures not only affected the number of persons attending the health facilities on the days of closure but also led to greater fear of infection at healthcare facilities and, in 1 instance, rumors that the clinic was closed for a longer period than it was (Table 2). Finally, health facility worker strikes and sit-ins over risk allowance payments and lack of personal protective equipment (PPE) also resulted in temporary closures of facilities (Table 2).

#### Effects of COVID-19 Prevention Measures on Healthcare Access

Government COVID-19 prevention measures that required use of facemasks and social distancing also were reported to have contributed to reduced access to health services. Mandatory use of face masks at health facilities was introduced during the epidemic, but TB officers cited the inability to afford a mask and the feeling that masks “suffocate them” as reasons patients did not want to wear masks (Table 2). Patients who tried to attend facilities without having a mask were sent away (meaning that they were not seen by a healthcare worker) and often did not return (Table 2). Public transportation in Blantyre also had a limit on vehicle capacity, which led to doubled transport costs and limited clinic access (Table 2).

#### TB-Specific Issues

Because TB and COVID-19 both have symptoms of cough and fever, TB officers reported issues around TB testing. First, persons with fever and cough reportedly were afraid of being tested for COVID-19 if they went to healthcare facilities. TB officers said patients were more afraid of COVID-19 than TB because they knew that TB could be cured and that patients with COVID-19 might need to be placed under facility isolation (Table 2). The similarity of symptoms also led to persons who normally would have been tested for TB being turned away from healthcare facilities and told to go home and call the COVID-19 help line (Table 2).

#### Reduced Healthcare Worker Capacity

TB officers also spoke of their own fear of contracting COVID-19 from presumptive TB patients. TB officers reported changing how they interacted with symptomatic persons, including interacting less directly and not supervising sputum collection as closely (Table 2). In addition, many TB officers reported that the lack of PPE in health facilities forced them to temporarily stop conducting TB tests or supervising sputum collection at all. For those patients who did submit sputum, results could be delayed because, as a TB officer reported, laboratory staff “were taught that sputum has the highest concentration of COVID-19” (Table 2).

## Discussion

In addition to directly causing millions of deaths, the COVID-19 pandemic has directly and indirectly affected delivery of health services globally (*14*). In our analysis of the effects of the COVID-19 pandemic on TB notifications in Blantyre, Malawi, we found a substantial immediate decline in TB case notifications concurrent with the start of the COVID-19 epidemic in Malawi. Our findings are consistent with initial reports on COVID-19 effects on HIV and TB diagnosis and care from other settings (*15*–*22*). However, we show that, after an initial decline, TB CNRs increased and reached near prepandemic levels within 9 months. Overall, we estimate that 333 fewer cases of TB were notified, equivalent to 39 cases/100,000 persons, during April–December 2020 than would have been expected in the absence of the COVID-19 epidemic. For the affected persons, the missed or delayed diagnoses likely will have severe consequences, and for public health programs the consequences might hinder progress toward TB elimination. The reduction in TB case notifications also could be indicative of more general disruption of a range of primary healthcare services.

To put these results into context, Malawi has high HIV and TB burdens. Estimated prevalence of TB in urban Malawi was 988 cases/100,000 persons at the last national survey in 2013 (*4*). TB in Malawi is declining in response to concerted efforts from the national and district TB and the HIV programs. In June 2016, Malawi introduced a test-and-treat program for HIV, which involved starting antiretroviral therapy for persons who had positive HIV tests regardless of CD4 cell count. Malawi is coming close to achieving United Nations AIDS/HIV 90-90-90 goals (*23*). However, TB remains one of the leading causes of death and years of life lost in Malawi (*24*).

We hypothesize that the major reason for the drop in TB notifications during the COVID-19 pandemic is that persons with true TB disease had their TB diagnosis missed or at least delayed. This hypothesis is consistent with data from our qualitative interviews with TB officers, who noted that, in the immediate period after the Malawi COVID-19 epidemic began, access to health facilities was extremely challenging. Alternative explanations are that persons with diagnosed TB started on treatment, but their cases were not notified to the national program, or that the true incidence of TB declined. However, we consider these explanations unlikely. TB treatment cannot be accessed in Malawi outside of TB registration centers, and our electronic TB surveillance system is cross-referenced with paper ledgers that confirm the same trends in notifications. Reduced incidence of other respiratory pathogens, notably influenza, has resulted from the nonpharmaceutical interventions for COVID-19, which possibly also resulted in a decline in TB transmission. However, the prolonged interval between infection and onset of symptoms for TB makes an immediate effect on notifications in <3 months implausible, particularly because Malawi has had less stringent COVID-19 prevention measures than many other countries.

Our qualitative interviews indicate that, in addition to general restrictions on healthcare access during the COVID-19 epidemic, TB testing and notifications particularly were affected because of the similarity in clinical presentation of TB and COVID-19. The TB officers considered that persons with TB symptoms were less likely to attend facilities for fear of a COVID-19 diagnosis and possible consequences, such as isolation. In addition, TB officers believed that at least some persons with possible TB who went to healthcare facilities were turned away and directed to COVID-19–specific services where they would be unlikely to be assessed for TB. In countries with high TB burdens, alignment of COVID-19 and TB diagnosis, prevention, and care will likely lead to improved outcomes for both diseases.

Women and girls had disproportionately higher reductions in case notifications than men and boys, as did HIV-negative compared with HIV-positive patients and notifications from primary care clinics compared with the central hospital. We hypothesize that women and girls faced greater barriers to accessing healthcare during COVID-19 than men and boys because of greater requirements of women to stay home to school children; social gender norms, meaning that men were more likely to disregard COVID-19 public health restrictions; and perhaps economic requirements for men leave the house to work, meaning men could more easily continue to access TB services (*25*).

Primary healthcare centers were more affected than QECH, both in terms of initial step change (drop in TB cases notified at the start of COVID-19) and with slower recovery in the period after the initial phase of COVID-19 epidemic in Malawi. Reasons for the difference in reporting rates could include QECH being prioritized for PPE, thus remaining more functional than healthcare centers; in addition, patients with TB diagnosed at QECH tend to have more severe illness and potentially were unable to delay seeking healthcare.

TB cases among HIV-negative persons declined more than among persons living with HIV, which also could be associated with site of TB diagnosis. QECH has the largest number of HIV-positive persons registered for antiretroviral therapy in the city, and so persons living with HIV may have accessed TB services through the ART clinic. Alternatively, persons living with HIV can have more severe TB symptoms and be less able to defer healthcare seeking.

Limitations to our study include uncertainty around the counterfactual conditions; during June 2016 –March 2020, TB case notifications were declining in Blantyre, and for the counterfactual condition, no COVID-19 scenario we modeled TB notifications as continuing to decline at the same rate. Since December 2020, Malawi has had a second wave of COVID-19. Our electronic enhanced surveillance data are entered in real time, but data are monitored and verified on a quarterly basis, so we do not yet have information on the effects of the second wave of COVID-19 in Malawi. Finally, we only interviewed healthcare workers; we did not directly capture perspectives of patients about their difficulties accessing healthcare.

Malawi is fortunate to have well-functioning TB and HIV programs that are more resilient to COVID-19 than programs in other countries. Malawi did not introduce any substantial restrictions on population movement and gathering due to COVID-19, so no legal restrictions hindered travel to TB clinics. Therefore, our data are not necessarily generalizable to other settings in southern Africa or elsewhere.

In conclusion, the effects of missed or delayed TB diagnoses likely will be severe for affected persons and households. However, the initial COVID-19–related decline in TB case notification was not sustained, and the Malawi National Tuberculosis Programme had a relatively quick recovery after the first wave of COVID-19. We observed a shorter period of disruption than earlier modeling of COVID-19 effects on TB assumed (*5*). COVID-19 or TB diagnosis, treatment, care, and public health measures should not be considered in isolation. Rather, public health and healthcare officials should seek opportunities to combine resources to tackle both COVID-19 and TB. Through improved infection prevention and control at health facilities, strengthened laboratory infrastructure, and community engagement to address stigma and provide sources of information about both diseases, communities can create a setting of universal health coverage.

Appendix Additional information on effects of the COVID-19 epidemic on TB notifications in Malawi.
