# Supplementary material for: Effects of Coronavirus Disease Pandemic on Tuberculosis Notifications, Malawi
Source: Emerg Infect Dis. 2021 Jul;27(7):1831–9. doi: 10.3201/eid2707.210557 (PMC8237899; doi:10.3201/eid2707.210557)
Supplement: Appendix — Additional information on effects of the COVID-19 epidemic on TB notifications in Malawi. [file 21-0557-Techapp-s1.pdf]

Article DOI: https://doi.org/10.3201/eid2707.210557

Effects of Coronavirus Disease Pandemic on Tuberculosis Notifications, Malawi

Appendix

Models

We used 2 models to estimate effects of coronavirus disease (COVID-19) on rates of tuberculosis (TB) case notifications in Blantyre, Malawi. One model estimates the effects of COVID-19 on TB case notifications, the other estimates differential effects of COVID-19 on TB case notifications by various factors.

Model 1

To estimate effects of COVID-19 on Blantyre TB case notification rates we used the following formula:

$$\log(\text{cases}) = \alpha + \beta_1(\text{covid}) + \beta_2(\text{month}) + \beta_3(\text{covid} \times \text{month.since.covid}) + \log(\text{population})$$

In this formula, cases are the expected numbers of notified TB cases per month, α is the intercept, covid is the indicator variable for the period before or after introduction of COVID-19 restrictions (before or after April 1, 2020), month is the indicator for month from June 2016, month.since.covid is the number of months after April 1, 2020, and population is the monthly updated population denominator for Blantyre District.

Model 2

To estimate differential effects of COVID-19 on the number of TB cases notified by sex, HIV status, and whether TB was diagnosed at a primary care versus central hospital, we used the following formula:

$$\log(\text{cases}) = \alpha + \beta_1(\text{sex}_{\text{male}}) + \beta_2(\text{hiv}_{\text{positive}}) + \beta_3(\text{fac}_{\text{qech}}) + \sum_{ijk} \beta_{ijk}^{(0)}(\text{sex}_i \times \text{hiv}_j \times \text{fac}_k)\ \text{month} + \text{covid} \times \sum_{ijk} \beta_{ijk}^{(1)}(\text{sex}_i \times \text{hiv}_j \times \text{fac}_k)\text{month.since.covid}$$

In this formula, cases indicates the expected numbers of notified TB cases per month, α is the intercept, covid is the indicator variable for the period before or after introduction of COVID-19 restrictions (before or after April 1, 2020), month is the indicator for month from June 2016, month.since.covid is the number of months after April 1, 2020, sex_i is the indicator for the sex of a person (i = positive, $\in$ = negative]), hiv_j is the indicator for HIV status of person (j = male, $\in$ = female]), fac_k is the indicator for the facility where TB was diagnosed (k = hc, $\in$ = qech] in which hc indicates a primary healthcare center and qech indicates Queen Elizabeth Central Hospital).
